# An Essential Physiological Role for MCT8 in Bone in Male Mice

**DOI:** 10.1210/en.2017-00399

**Published:** 2017-06-15

**Authors:** Victoria D. Leitch, Caterina Di Cosmo, Xiao-Hui Liao, Sam O’Boy, Thomas M. Galliford, Holly Evans, Peter I. Croucher, Alan Boyde, Alexandra Dumitrescu, Roy E. Weiss, Samuel Refetoff, Graham R. Williams, J. H. Duncan Bassett

**Affiliations:** 1 Molecular Endocrinology Laboratory, Department of Medicine, Hammersmith Campus, Imperial College London, London W12 0NN, United Kingdom; 2 Department of Medicine, The University of Chicago, Chicago, Illinois 60637; 3 Sheffield Myeloma Research Team, University of Sheffield, Sheffield S10 2RX, United Kingdom; 4 The Garvan Institute of Medical Research and St. Vincent’s Clinical School, University of New South Wales Medicine, Sydney, New South Wales 2010, Australia; 5 Queen Mary University of London, Oral Growth and Development, Bart’s and The London School of Medicine and Dentistry, London E1 4NS, United Kingdom; 6 Department of Medicine, University of Miami, Miami, Florida 33136; 7 Department of Pediatrics, The University of Chicago, Chicago, Illinois 60637; 8 Committee on Genetics, The University of Chicago, Chicago, Illinois 60637

## Abstract

T3 is an important regulator of skeletal development and adult bone maintenance. Thyroid hormone action requires efficient transport of T4 and T3 into target cells. We hypothesized that monocarboxylate transporter (MCT) 8, encoded by *Mct8* on the X-chromosome, is an essential thyroid hormone transporter in bone. To test this hypothesis, we determined the juvenile and adult skeletal phenotypes of male *Mct8* knockout mice (*Mct8KO*) and *Mct8D1D2KO* compound mutants, which additionally lack the ability to convert the prohormone T4 to the active hormone T3. Prenatal skeletal development was normal in both *Mct8KO* and *Mct8D1D2KO* mice, whereas postnatal endochondral ossification and linear growth were delayed in both *Mct8KO* and *Mct8D1D2KO* mice. Furthermore, bone mass and mineralization were decreased in adult *Mct8KO* and *Mct8D1D2KO* mice, and compound mutants also had reduced bone strength. Delayed bone development and maturation in *Mct8KO* and *Mct8D1D2KO* mice is consistent with decreased thyroid hormone action in growth plate chondrocytes despite elevated serum T3 concentrations, whereas low bone mass and osteoporosis reflects increased thyroid hormone action in adult bone due to elevated systemic T3 levels. These studies identify an essential physiological requirement for MCT8 in chondrocytes, and demonstrate a role for additional transporters in other skeletal cells during adult bone maintenance.

## Teaser

Studies of KO mice demonstrate an essential role for the thyroid hormone transporter MCT8 in chondrocytes during skeletal development and reveal the importance of other transporters in adult bone maintenance.

Thyroid hormone is an important regulator of skeletal development, linear growth, and adult bone mass and strength (1). The prohormone T4 (3,5,3′,5′-l-tetraiodothyronine, thyroxine) is the most abundant circulating thyroid hormone, but it must be metabolized to the active hormone T3 (3,5,3′-l-triiodothyronine) for physiological activity (2). The major effects of T3 in bone are mediated by thyroid hormone receptor *α*1, but these actions are dependent on efficient transport and intracellular metabolism (1). Entry of T4 and T3 into target tissues is facilitated by cell membrane transporters including the l-type amino acid transporters 1 and 2, organic anion transporter polypeptide-1c1, and monocarboxylate transporters (MCTs) 8 and 10 (3).

The type 1 and 2 iodothyronine deiodinases (DIO1 and DIO2) convert T4 to T3 by removal of an outer-ring iodine atom. A third enzyme (DIO3) prevents activation of T4 and inactivates T3 by inner-ring deiodination, generating the metabolites reverse T3 (3,3′,5′-triiodothyronine; rT3) and T2 (3,3′-diiodothyronine), respectively (2). DIO1 is not expressed in the skeleton, whereas the relative activities of DIO2 and DIO3 determine the intracellular availability of T3 in target tissues including bone (4, 5). DIO3 is expressed most abundantly during intrauterine development when it protects developing tissues from premature exposure to T3, whereas levels of DIO2 rise mainly from birth (1, 2, 6). Expression of DIO2 in the epiphyseal growth plate regulates T3 availability and the pace of chondrocyte differentiation during early skeletal development (6, 7), and its expression in bone-forming osteoblasts controls T3 regulation of adult bone mineralization and strength (8).

In contrast to the established importance of DIO2 and DIO3 in the regulation of T3 action in bone, it is not known which transporters facilitate thyroid hormone entry into skeletal cells. l-type amino acid transporters 1 and 2 are expressed in the skeleton, but their lack of response to altered thyroid hormone concentrations, and their relatively lower affinity and specificity for T4 and T3, suggest they do not have a major role in control of T3 action in bone (4, 9, 10). Although organic anion transporter polypeptide-1c1 is a high-affinity transporter for T4, it is not expressed in skeletal cells (10). By contrast, MCT8 is expressed and regulated by thyroid hormone in growth plate chondrocytes, bone-forming osteoblasts, and bone-resorbing osteoclasts (4, 10), whereas MCT10 is expressed in chondrocytes (9) but has not been studied in other skeletal cells. Mutations of the *MCT8* gene in humans (OMIM 300523) cause severe X-linked psychomotor retardation together with high serum T3, elevated or normal thyrotropin (TSH), decreased T4, and markedly reduced rT3 concentrations (11–13). Although linear growth is not significantly impaired in the majority of cases, bone age has been reported as normal (11, 12, 14), advanced (15), or delayed (16) in individual patients. Deletion of *Mct8* in mice also results in increased T3 and TSH levels and reduced serum T4 and rT3 concentrations but fails to recapitulate the psychomotor retardation (17, 18), whereas effects on the skeleton have not been studied.

We hypothesized that MCT8 is an essential thyroid hormone transporter required for bone development, mineralization, and strength. To test this hypothesis, we determined the skeletal consequences of deletion of *Mct8* in *Mct8*^−/y^ knockout (KO) mice (*Mct8KO*) compared with wild-type (WT) mice. To investigate the possibility that other thyroid hormone transporters could contribute to thyroid hormone uptake, we also compared *Mct8KO* mice to triple KO *Mct8*^−/y^*Dio1*^−/−^*Dio2*^−/−^ mice, which are deprived of the intracellularly generated active T3 as they lack both thyroid hormone–activating enzymes and *Mct8* (*Mct8D1D2KO*). In these studies, an abnormal skeletal phenotype in *Mct8KO* mice would indicate an essential physiological role for MCT8 in bone. An equivalent phenotype in *Mct8D1D2KO* mice would identify MCT8 as the sole thyroid hormone transporter in the skeleton, whereas a more severe skeletal phenotype in *Mct8D1D2KO* mice would suggest an additional transporter contributes to thyroid hormone uptake in bone.

## Materials and Methods

### Mice

*Dio1* KO (*Dio1*^−/−^, *D1KO*), *Dio2* KO (*Dio2*^−/−^, *D2KO*) and *Mct8* KO (*Mct8*^−/y^, *Mct8KO*) mice have been described previously and were crossed to obtain *Mct8KO*, *Mct8*^−^*^/y^Dio1*^−/−^*Dio2*^−/−^ (*Mct8D1D2KO*) and WT mice (19). All KO mice were backcrossed more than 10 times with the WT C57BL/6 strain. Mice were housed at 22°C ± 2°C with a 12-hour light/12-hour dark cycle, and access to Purina Rodent Chow (0.8 ppm iodine; Purina Mills, St. Louis, MO) and water *ad libitum*. Male mice were collected at postnatal days (P)1, P14, P32, P77, and P112. Adult mice were given intraperitoneal injections of calcein (10 mg/kg in 100 µL phosphate-buffered saline) 14 and 7 days before euthanasia (20).

### Ethics

Animal studies were performed according to a protocol approved following independent review by The University of Chicago Institutional Animal Care and Use Committee.

### Hormone levels

Circulating levels of total T3, T4, rT3, and TSH were measured by radioimmunoassay in serum samples collected at P1 (WT, n = 4; *Mct8KO*, n = 5; *Mct8D1D2KO*, n = 4), P14 (WT, n = 4; *Mct8KO*, n = 4; *Mct8D1D2KO*, n = 4), P32 (WT, n = 4; *Mct8KO*, n = 3; *Mct8D1D2KO*, n = 3), P77 (WT, n = 4; *Mct8KO*, n = 4; *Mct8D1D2KO*, n = 4), and P112 (WT, n = 4; *Mct8KO*, n = 5; *Mct8D1D2KO*, n = 5) as described (21).

### Whole mount stains

P1 mice were euthanized, fixed, and stored in 70% ethanol. Skin and viscera were removed, and the intact skeleton stained with alizarin red and alcian blue and stored in 100% glycerol (22). Stained P1 mice were imaged using a Leica MZ75 binocular microscope, KL1500 light source, DFC320 digital camera, and IM50 Digital Image Manager (Leica Microsystems, Heerbrugg, Switzerland).

### RNA isolation and quantitative reverse transcription polymerase chain reaction

Whole tibias from WT mice between embryonic day (E) 14.5 (E14.5) and P186 were pulverized at −80°C using a steel pestle and mortar (Biospec; Thistle Scientific, Glasgow, Scotland, UK), the resulting powder was homogenized in TRIzol (Thermo Fisher Scientific, Waltham, MA), and RNA was extracted (n = 8 per age). Quantitative reverse transcription polymerase chain reaction (qRT-PCR) was performed using complementary DNA synthesized with polyA primers and superscript II reverse transcription (Thermo Fisher Scientific). A total of 1 μg RNA was denatured at 70°C for 10 minutes, and polyA primers and 1 U of superscriptase II were added and incubated for a further 30 minutes at 42°C. Standard PCR was performed using Platinum Taq DNA polymerase (Thermo Fisher Scientific) to optimize conditions for quantitative polymerase chain reaction (qPCR). Expression of *Mct8* was determined by qPCR after RNA quantity and quality was confirmed using a Nanodrop 1000 spectrophotometer (Thermo Fisher Scientific). Seven hundred fifty nanograms RNA was converted to complementary DNA using a Quantitect reverse transcription kit (QIAGEN, Manchester, UK) and used for qRT-PCR using a KAPA SYBR Fast qPCR kit (KAPA Biosystems, London, UK). Reactions were run on a 7900HT real-time PCR system (Thermo Fisher Scientific) for between 30 and 40 cycles (primers in Table 1). Samples were run in duplicate and results calculated by comparison with a standard curve and normalized relative to expression of *Gapdh*.

**Table 1.** **qRT-PCR Primers**

**Gene**	**MGI Symbol**	**Accession No.**	**Ensemble Transcript ID**	**Primer Sequence (5′–3′)**	**T_m_, °C**	**Size, bp**
*Mct8*	*Mct8*	NM_009197.2	ENSMUST00000042664	F: TTGCTTTCATTGGCCTCCA	52	143
	*(Slc16a2)*			R: GCGACGTTGAAAGTAGTGGC		
*Gapdh*	*Gapdh*	NM_008084	ENSMUST00000073605	F: AACTTTGGCATTGTGGAAGG	54	223
				R: ACACATTGGGGGTAGGAACA		

Abbreviations: bp, base pair; F, forward; MGI, Mouse Genome Informatics; R, reverse.

### Histology

Lower limbs were fixed in 10% neutral buffered formalin for 24 hours and decalcified using 10% EDTA pH 7.4. Decalcification was verified by digital X-ray microradiography (MX20 Faxitron: Qados, Cross Technologies plc, Sandhurst, Berkshire, UK). Paraffin-embedded 5-μm sections were stained with alcian blue and van Gieson (8, 20) and imaged using a Leica DM LB2 microscope and Leica DFC320 digital camera. Total growth plate height and growth plate zone measurements were determined at a minimum of four separate positions using ImageJ (http://rsb.info-nih.gov/ij/) to determine mean values. Results from two levels of sectioning were compared (23).

### Digital X-ray microradiography

Upper limbs, lower limbs, and tail vertebrae were imaged using a Faxitron MX20 at 26 kV, and ×5 projective magnification giving 10-μm resolution, and bone mineral content (BMC) relative to steel, aluminum, and polyester standards was determined. Images were calibrated with a digital micrometer, and bone length, cortical bone diameter, and thickness were determined (8, 23, 24).

### Microcomputerized tomography

Femurs were imaged in 70% ethanol using a Skyscan 1172a microcomputerized tomography scanner (Bruker MicroCT, Kontich, Belgium). Scans were performed at 50 kV, 200 μA, 0.5-mm aluminum filter with a detection pixel size of 4 μm^2^, and images were reconstructed using Skyscan NRecon software. Trabecular number (Tb.N), thickness (Tb.Th), bone volume as a proportion of tissue volume (BV/TV), and structure model index were calculated within a 1-mm^3^ region of interest located 0.2 mm below the growth plate (8, 20).

### Three-dimensional backscattered electron-scanning electron microscopy

Femurs and tibia were opened longitudinally and macerated as described (25). Carbon-coated samples were imaged using backscattered electrons with a Zeiss DSM962 digital scanning electron microscope (Carl Zeiss Ltd., Cambridge, UK) at 20 kV beam potential. The fraction of trabecular and endosteal bone surfaces displaying osteoclastic resorption were quantified in high-resolution images using ImageJ (8).

### Quantitative backscattered electron-scanning electron microscopy

Neutral buffered formalin–fixed humeri and tibias were embedded in methacrylate. Longitudinal block faces were cut through specimens, which were then polished, coated with carbon, and analyzed using backscattered electrons at 20 kV, 0.5 nA with a working distance of 17 mm and a sample-to-detector distance of 11 mm. Bone mineralization densities were determined by comparison with halogenated dimethacrylate standards, and an eight-interval pseudocolor scheme was used to represent the graduations of micromineralization (8, 25, 26).

### Static osteoclast histomorphometry

Sections from decalcified tibias were stained for tartrate-resistant acid phosphatase and imaged using a Leica DM LB2 microscope and DFC320 digital camera (8, 23). A montage of nine overlapping fields covering an area of 1 mm^2^ located 0.2 mm below the growth plate was constructed for each bone. BV/TV was measured, and osteoclast numbers and surface were determined in trabecular bone normalized to total bone surface (23).

### Dynamic osteoblast histomorphometry

Methacrylate-embedded calcein-labeled tibias were imaged with a Leica SP2 reflection confocal microscope at 488-nm excitation. Parameters of bone formation were determined using ImageJ according to the American Society for Bone and Mineral Research system of nomenclature (27, 28). The mineral apposition rate (MAR) was calculated by determining calcein separation at 20 locations per specimen beginning 0.2 mm below the growth plate and including both cortical and trabecular surfaces. Bone formation rate (BFR) was calculated from the product of mineralizing surface (MS) and MAR (8, 23, 28).

### Destructive three-point bend testing

Tibias were stored and tested in 70% ethanol. Mechanical strength was determined by destructive three-point bend testing using an Instron 5543 load frame with a 100-N load cell and a constant rate of displacement of 0.03 mm/s until fracture (Instron Limited, High Wycombe, Buckinghamshire, UK). Biomechanical variables were calculated from load displacement curves (8, 29).

### Statistical analysis

Data are shown as mean ± standard deviation unless otherwise indicated. Normally distributed data were analyzed by analysis of variance followed by Tukey *post hoc* test. *P* values < 0.05 were considered significant. Frequency distributions of mineralization densities from quantitative backscattered electron-scanning electron microscopy (BSE-SEM) and digital X-ray microradiography images were compared using the Kolmogorov-Smirnov test (8, 20, 24).

## Results

### Thyroid dysfunction in Mct8KO and Mct8D1D2KO mice

**Figure 1.**
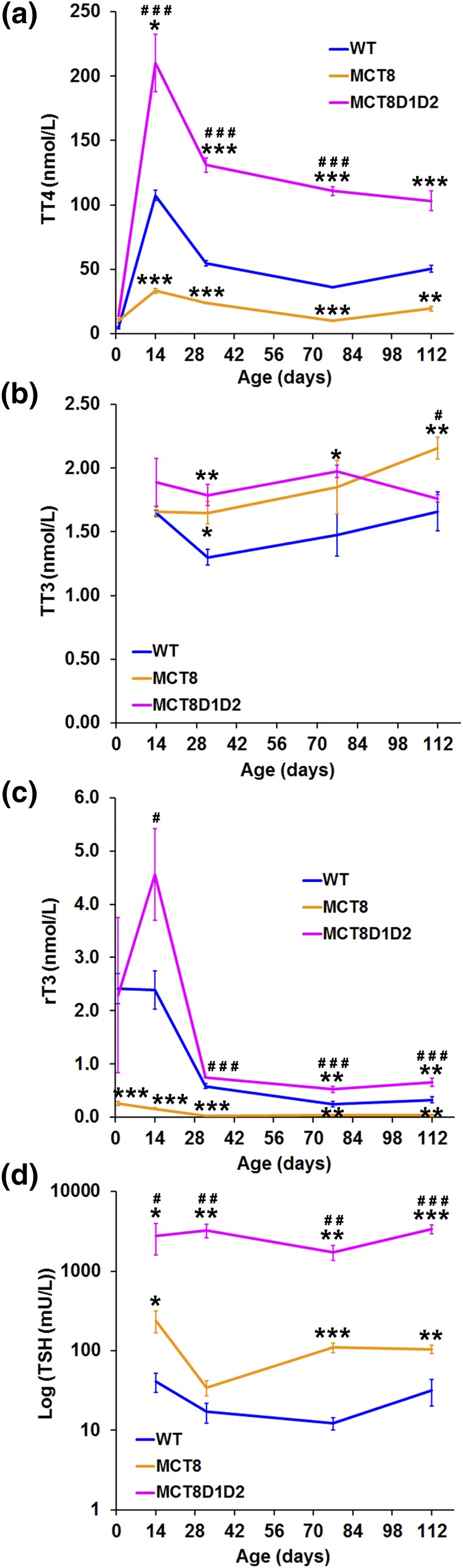
Serum thyroid hormone levels. (a) Total T4 and (c) rT3 levels in P1, P14, P32, P77, and P112 mice, and (b) total T3 and (d) TSH in P14, P32, P77, and P112 mice. Data are mean ± standard error of the mean; n = 3–5 per genotype per age; *P < 0.05, **P < 0.01, ***P < 0.001 vs WT; #*P* < 0.05, ##*P* < 0.01, ###*P* < 0.001 vs *Mct8KO*; analysis of variance followed by Tukey *post hoc* test.

*Mct8KO* mice had decreased T4 and rT3 levels but increased TSH and slightly elevated T3 concentrations compared with WT mice (Fig. 1), as reported previously (17–19, 30). Consistent with previous data (19), *Mct8D1D2KO* mice had elevated T4, rT3, and TSH concentrations at all ages compared with WT and *Mct8KO* mice. Serum T3 levels were also elevated in *Mct8D1D2KO* mice compared with WT but not *Mct8KO* mice. Overall, *Mct8KO* mice have mild central resistance to thyroid hormone with decreased T4 concentrations and slightly elevated T3 concentrations leading to an increased systemic T3:T4 ratio. These abnormalities are accompanied by decreased DIO3-mediated 5-deiodination but increased DIO1- and DIO2-mediated 5′-deiodination (19). By contrast, *Mct8D1D2KO* mice have severe central resistance to thyroid hormone with systemic hyperthyroidism and increased 5-deiodination but absent 5′-deiodination.

### Normal prenatal skeletal development in Mct8KO and Mct8D1D2KO mice

**Figure 2.**
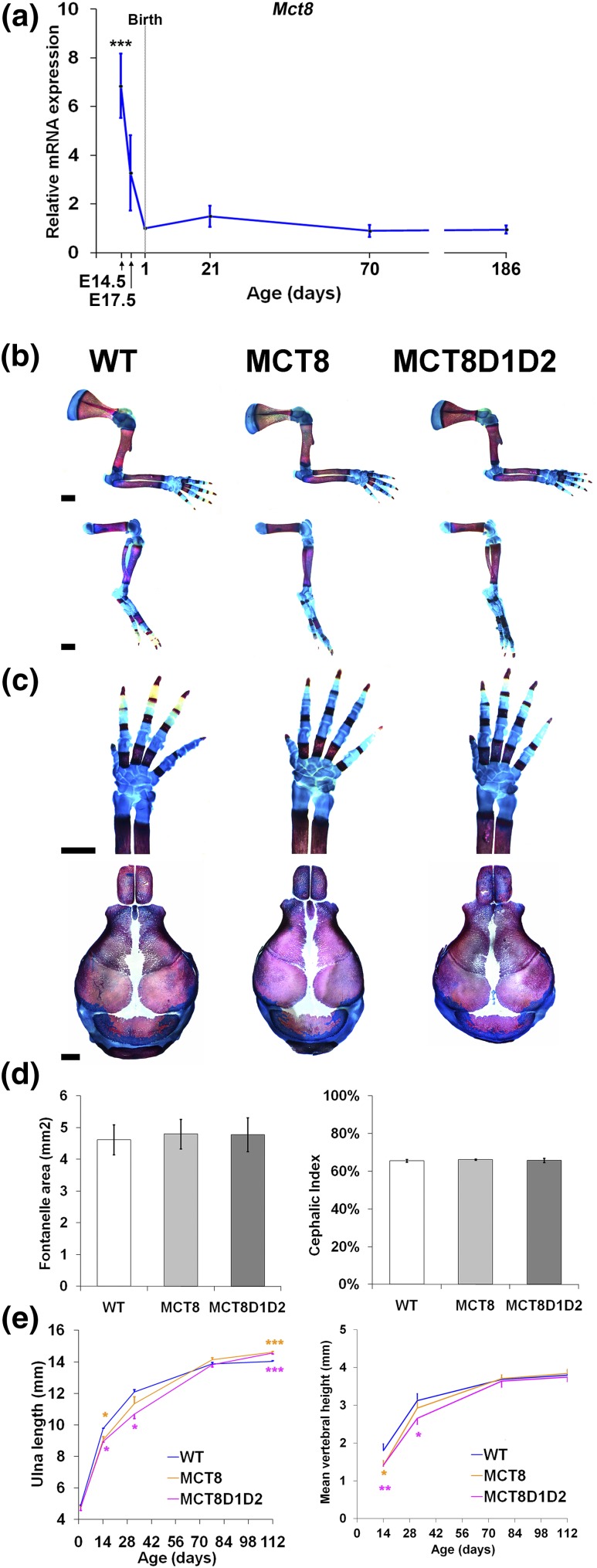
Skeletal development and linear growth. (a) *Mct8* mRNA expression in whole tibias from WT mice (n = 8 biological replicates per group, 2 technical replicates per sample); analysis of variance (ANOVA) , two-sided Tukey *post hoc* test; ***P < 0.001 vs expression at P1. (b) Limbs from P1 WT, *Mct8KO*, and *Mct8D1D2KO* mice stained with alizarin red (bone) and alcian blue (cartilage); scale bar = 1 mm. (c) Forelimb digits and cranial vaults stained with alizarin red and alcian blue; scale bar = 1 mm. (d) Fontanelle area and cephalic index (cephalic index is calculated by dividing cranial width by cranial length and then multiplying the result by 100) in P1 skulls, n = 4 per genotype. (e) Ulna lengths and caudal vertebra heights from birth to P112.Data are mean ± standard error of the mean; n = 4 per genotype per age; *P < 0.05, **P < 0.01, ***P < 0.001 vs WT; ANOVA followed by Tukey *post hoc* test.

In WT mice, the normal physiological expression of *Mct8* in the skeleton was highest in the prenatal period but decreased thereafter and remained at a constant level in juvenile and adult WT mice [Fig. 2(a)]. Formation of ossification centers in the limbs did not differ between neonatal WT, *Mct8KO*, and *Mct8D1D2KO* mice at P1. Similarly, there were no differences in development of the skull [Fig. 2(b–d)]. Thus, prenatal endochondral and intramembranous ossification were normal in both *Mct8KO* and *Mct8D1D2KO* mice.

### Delayed postnatal endochondral ossification and linear growth in Mct8KO and Mct8D1D2KO mice

**Figure 3.**
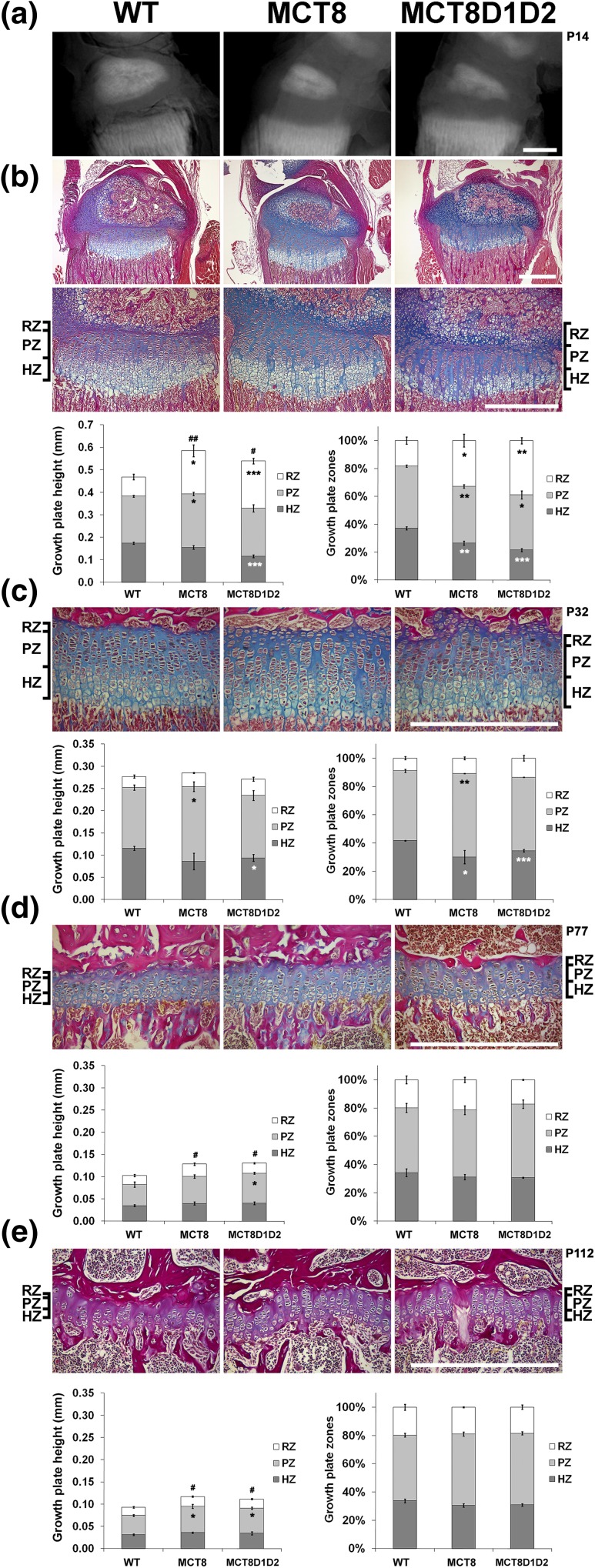
Endochondral ossification. (a) Microradiographs of proximal tibia from P14 WT, *Mct8KO*, and *Mct8D1D2KO* mice; scale bar = 0.5 mm. (b) Decalcified sections of P14 proximal tibia stained with alcian blue (cartilage) and van Gieson (bone matrix); scale bars = 0.5 mm. The left graph shows growth plate, RZ, PZ, and HZ heights. The graph on the right shows relative values, where each zone is shown as a percentage of total growth plate height. (c–e) Proximal tibia sections from P32, P77, and P112 mice with graphs showing total and relative growth plate zone heights. Data are mean ± standard error of the mean; n = 4 per genotype; *P < 0.05, **P < 0.01, ***P < 0.001 vs height of zone in WT; #*P* < 0.05, ##*P* < 0.01 vs total growth plate height in WT; analysis of variance followed by Tukey *post hoc* test; scale bars = 0.5 mm.

*Mct8KO* and *Mct8D1D2KO* mice had similar degrees of postnatal growth retardation of long bones and vertebrae between P1 and P77 although long bone length was increased at P112 [Fig. 2(e)]. Accordingly, X-ray and histological analysis demonstrated delayed postnatal endochondral ossification in both *Mct8KO* and *Mct8D1D2KO* mice characterized by decreased secondary ossification center size and increased growth plate height compared with WT (Fig. 3). At P14 the increased height of the growth plate resulted mainly from an increase in the reserve zone (RZ), which was accompanied by a small increase in the proliferative zone (PZ) but a reduction in the hypertrophic zone (HZ). The height of the growth plate did not differ at P32 in either *Mct8KO* or *Mct8D1D2KO* mice compared with WT, although minor differences in the PZ and HZ relative to the total height of the growth plate were observed. The height of the growth plates remained increased at P77 and P112 in both *Mct8KO* and *Mct8D1D2KO* mice compared with WT, although the relative heights of the RZ, PZ, and HZ were normal.

Overall, these data are consistent with a similar degree of delayed endochondral ossification in *Mct8KO* and *Mct8D1D2KO* mice that results primarily from impaired recruitment of RZ chondrocyte progenitors to the PZ at P14 and leads to delayed postnatal growth. Delayed progression of chondrocytes through the PZ and HZ chondrocytes continues and persists into adulthood, resulting in continued linear growth between P77 and P112. These findings are characteristic of impaired T3 action in growth plate chondrocytes and the response of the developing skeleton to hypothyroidism (1).

### Abnormal BMC in Mct8KO and Mct8D1D2KO mice

**Figure 4.**
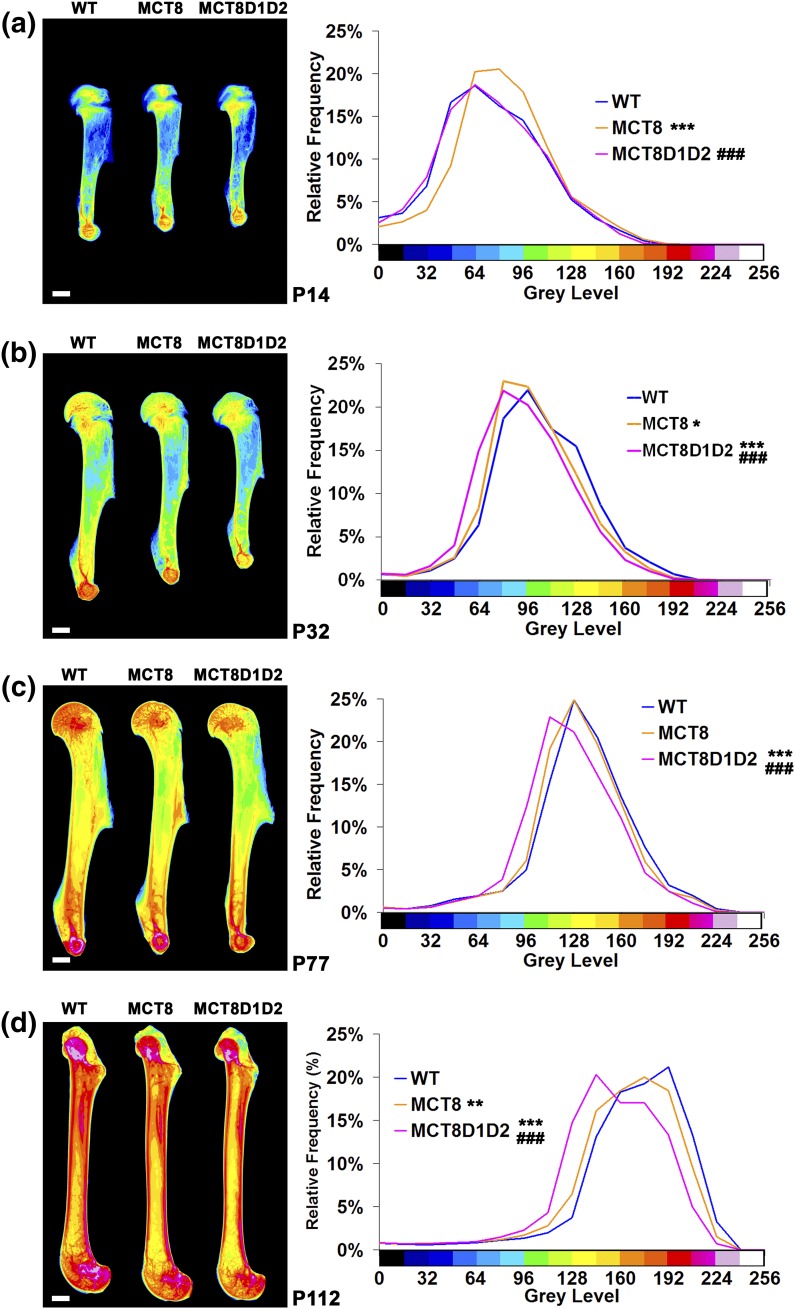
Bone mineral content. Quantitative microradiographic images of long bones from (a) P14, (b) P32, (c) P77, and (d) P112 WT, *Mct8KO* and *Mct8D1D2KO* mice; scale bars = 1 mm. Pseudocolored images represent grayscale images using a 16-color interval scheme with low mineral content in blue and high mineral content in pink. Relative frequency histograms of BMC (n = 4 per genotype per age). *P < 0.05, **P < 0.01, ***P < 0.001 vs WT, ###*P* < 0.001 vs *Mct8KO*; Kolmogorov-Smirnov test.

Long bones from *Mct8KO* mice had increased BMC at P14 but decreased BMC at P32. At P77 BMC was normal, whereas at P112 BMC was decreased. At all ages, BMC in *Mct8D1D2KO* mice was decreased compared with BMC in *Mct8KO* mice and was decreased compared with WT mice at all ages from P32 (Fig. 4).

### Decreased bone mass and mineralization in Mct8KO and Mct8D1D2KO mice

**Figure 5.**
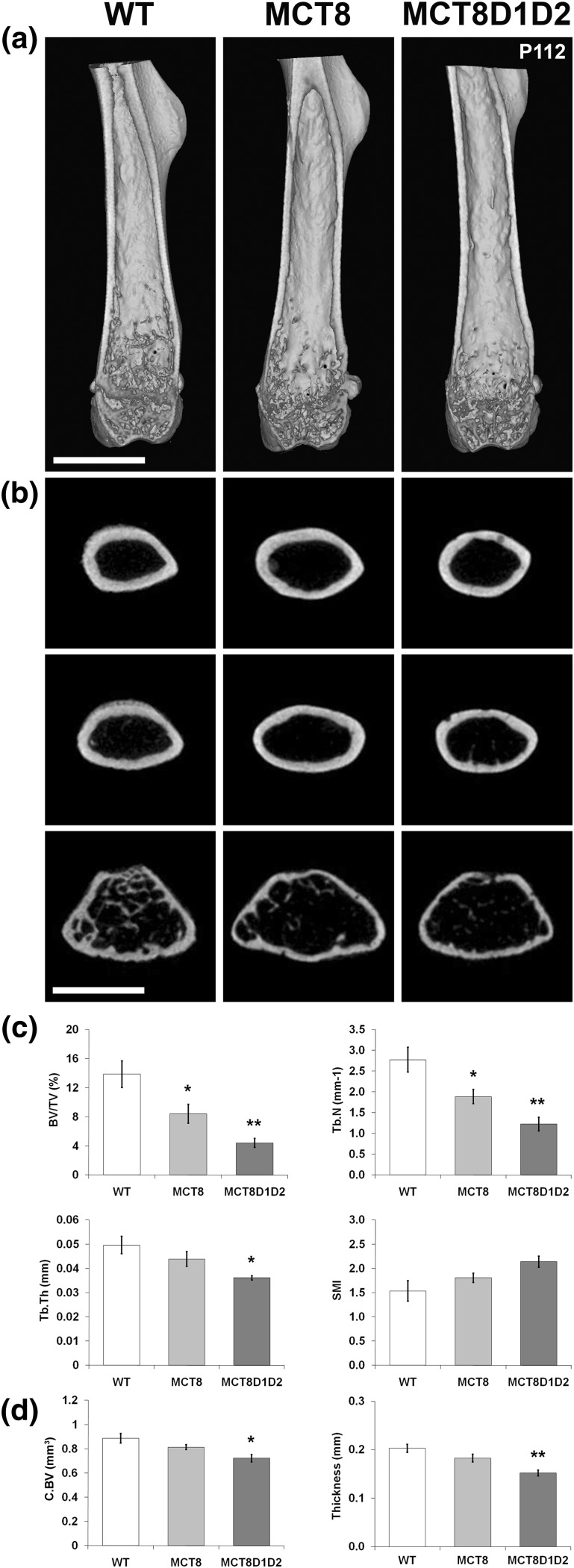
Bone structure. (a) Longitudinal midline microcomputerized tomography–rendered images of distal femur from P112 WT, *Mct8KO* and *Mct8D1D2KO* mice; scale bars = 2 mm. (b) Transverse views of proximal (upper panels), midshaft (middle panels), and distal (lower panels) femur; scale bars = 2 mm. (c) Trabecular bone structural parameters: BV/TV, Tb.N, Tb.Th, and structure model index (SMI). Data are mean ± standard error of the mean; n = 4 per genotype; *P < 0.05, **P < 0.01 vs WT; analysis of variance (ANOVA) followed by Tukey *post hoc* test. (d) Cortical bone structural parameters: cortical bone volume and cortical thickness. Data are mean ± standard error of the mean; n = 4 per genotype; *P < 0.05, **P < 0.01 vs WT; ANOVA followed by Tukey *post hoc* test.

**Figure 6.**
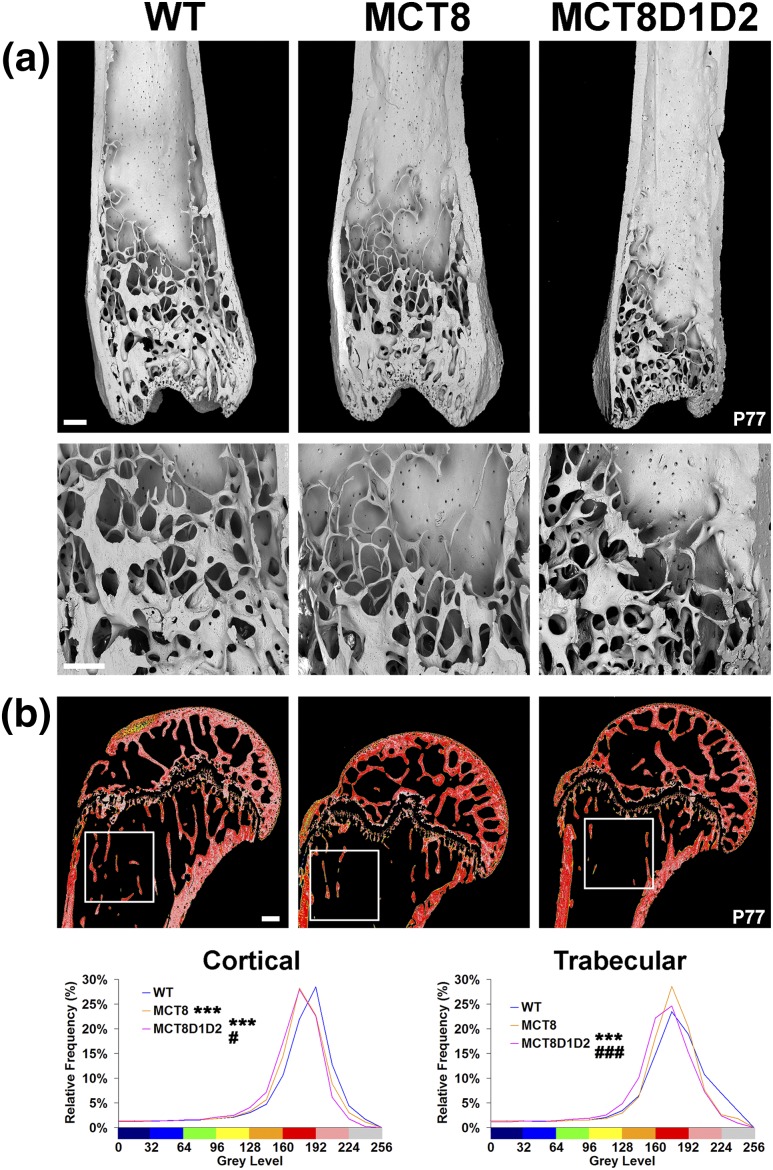
Bone microarchitecture and micromineralization density. (a) Low- and higher-power BSE-SEM images of distal femur from P77 WT, *Mct8KO*, and *Mct8D1D2KO* mice. Images are representative examples of n = 4 per genotype; scale bars = 200 μm. (b) Quantitative BSE-SEM images of proximal humerus from P77 WT, *Mct8KO*, and *Mct8D1D2KO* mice; scale bars = 200 μm. Grayscale images were pseudocolored using an eight-color interval scheme with low mineralization density in blue and high density in pink/white. White boxes indicate the region of interest used for quantitation of trabecular bone micromineralization density. Relative frequency histograms of bone micromineralization densities of proximal humerus and trabecular bone compartment. Images representative of n = 4 per genotype; ***P < 0.001 vs WT, #*P < 0.05* vs *Mct8KO*, ###*P* < 0.001 vs *Mct8KO*; Kolmogorov-Smirnov test.

Microcomputerized tomography analysis of the femur from P112 mice demonstrated decreased BV/TV and Tb.N in both *Mct8KO* and *Mct8D1D2KO* mice, with decreased Tb.Th and decreased cortical bone volume and thickness also seen in *Mct8D1D2KO* mice (Fig. 5). These findings were confirmed in P77 mice by BSE-SEM [Fig. 6(a)]. Further analysis by quantitative BSE-SEM demonstrated decreased bone mineralization density in both *Mct8KO* and *Mct8D1D2KO* mice, with *Mct8D1D2KO* mice more markedly affected [Fig. 6(b)]. Overall, adult *Mct8KO* and *Mct8D1D2KO* mice each had decreased bone mass and mineralization, with a more severe phenotype evident in *Mct8D1D2KO* animals.

### Increased trabecular bone resorption in Mct8D1D2KO mice

**Figure 7.**
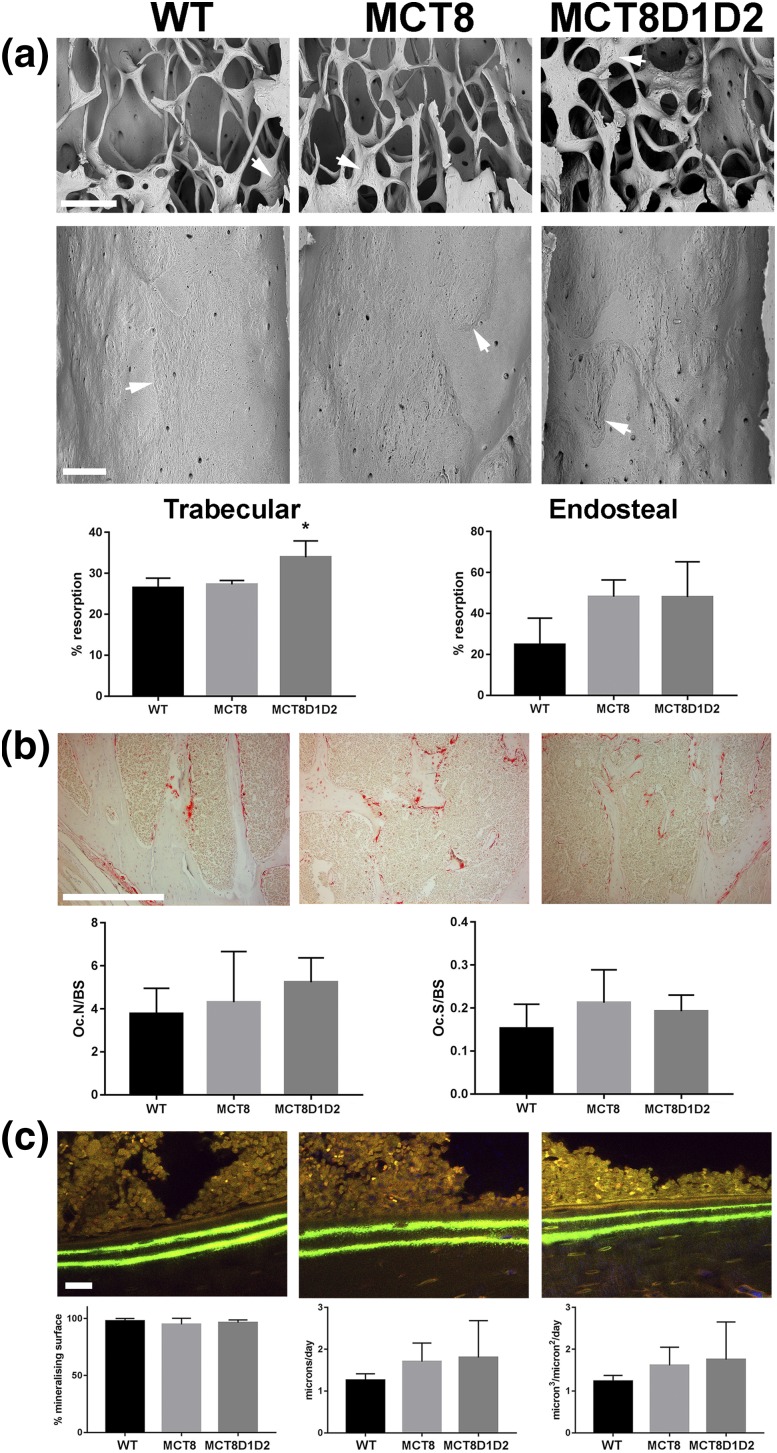
Osteoclastic bone resorption and osteoblastic bone formation. (a) Three-dimensional BSE-SEM images of trabecular bone and midfemur endocortical bone surface from femurs of P77 WT, *Mct8KO*, and *Mct8D1D2KO* mice (arrows indicate borders between regions of osteoclastic resorption and unresorbed bone surfaces, scale bars = 200 μm). Resorption surfaces shown as percentage of total bone surface in trabecular and cortical bone. Data are mean ± standard error of the mean; n = 4 per genotype; *P < 0.05 vs WT; analysis of variance followed by Tukey *post hoc* test. (b) Section from proximal humerus of P77 mice stained with tartrate-resistant acid phosphatase for osteoclasts in red; scale bar = 200 μm. Number of osteoclasts per millimeter of bone surface (OcN/BS) and osteoclast surface per millimeter of bone surface (OcS/BS). (c) Confocal images of humerus cortical bone double-labeled with calcein from P112 WT, *Mct8KO*, and *Mct8D1D2KO* mice; scale bars = 10 μm. Cortical MS, MAR, and BFR, n = 4 per genotype.

The underlying cellular basis for the observed defects in BMC and bone mass were investigated by static and dynamic histomorphometry. Trabecular bone resorption surfaces were increased in *Mct8D1D2KO* mice, but all other parameters of bone resorption [osteoclast number per bone surface and osteoclast surface per millimeter of bone surface formation (cortical MS, MAR, and BFR) were similar in WT, *Mct8KO*, and *Mct8D1D2KO* mice (Fig. 7). In summary, the greater decrease in bone mass observed in *Mct8D1D2KO* mice resulted from increased osteoclastic bone resorption.

### Decreased bone strength in Mct8D1D2KO mice

**Figure 8.**
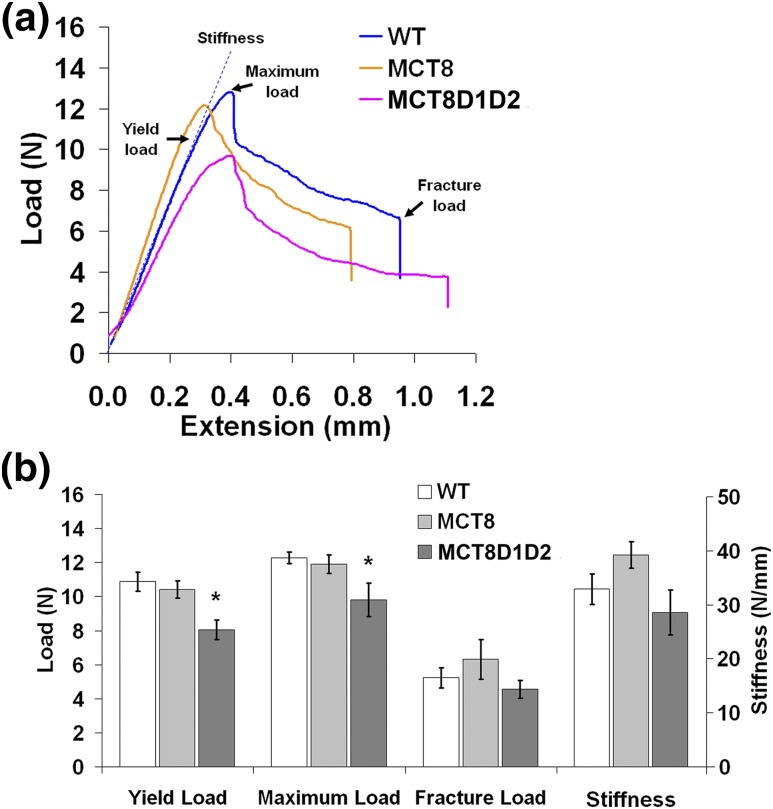
Bone strength. (a) Representative load displacement curves from three-point bend testing of P112 WT, *Mct8KO*, and *Mct8D1D2KO* tibias. (b) Yield, maximum and fracture loads, and stiffness. Data are mean ± standard error of the mean; n = 4 per genotype; *P < 0.05 vs WT; analysis of variance followed by Tukey *post hoc* test.

The consequences of the observed defects in BMC, mass, and mineralization on bone strength were determined in biomechanical studies. Tibias from adult *Mct8D1D2KO* mice were weak with decreased yield and maximum loads, whereas tibias from *Mct8KO* mice were of normal strength (Fig. 8).

## Discussion

The skeleton is an important T3-target tissue (Table 2) (1), and physiological thyroid hormone action requires efficient transport of T4 and T3 into target cells (3). We identified an essential role for MCT8 in the skeleton by analyzing (1) mice that lack *Mct8* alone (*Mct8KO*) and (2) mice that lack both *Mct8* and the ability to convert T4 to the active hormone T3 (*Mct8D1D2KO*).

**Table 2.** **Relationship Between Systemic Thyroid Status and Skeletal Phenotype in Various Mouse Models**

	**Serum Hormones**		**Juvenile Bone Development**	**Adult**		**Reference**
	**T4 Level**	**T3 Level**		**Bone Mass**	**Bone Strength**	
Hypothyroid	↓	↓	Delayed	↑	↑	Bassett and Williams (1)
Hyperthyroid	↑	↑	Advanced	↓	↓	Bassett and Williams (1)
D2KO	↑	N	N	↑	↑	Bassett *et al.* (8) and Galton *et al.* (31)
D1D2KO	↑	N	N	↑	↑	Bassett *et al.* (8) and Galton *et al.* (32)
MCT8KO	↓	↑	Delayed	↓	↓	Figs. 1–8
MCT8D1D2KO	↑	↑	Delayed	↓	↓	Figs. 1–8

Abbreviation: N, normal.

### Skeletal thyroid hormone deficiency and excess in Mct8KO and Mct8D1D2KO mice

In WT mice *Mct8* is expressed in the skeleton at high levels *in utero* but declines at birth and remains stable thereafter. During postnatal development, *Mct8KO* mice have delayed endochondral ossification and transient growth retardation accompanied by minor differences in BMC. Adult *Mct8KO* mice have decreased bone mass and mineralization, but bones are of normal strength. This complex phenotype was more severe in *Mct8D1D2KO* mice, in which adult osteoporosis resulted in decreased bone strength.

Thyroid hormone exerts anabolic actions during postnatal skeletal development but elicits catabolic responses in the adult skeleton (Table 2) (1). Thus, T3 stimulates bone mineral accrual during endochondral ossification and growth, but increases bone resorption in adults. In adults with thyrotoxicosis, increased bone turnover and uncoupling of bone resorption and formation results in accelerated bone loss, osteoporosis, and fracture (1). Thus, delayed bone development and maturation in *Mct8KO* and *Mct8D1D2KO* mice is consistent with skeletal thyroid hormone deficiency, whereas bone loss and osteoporosis in adults reflects skeletal thyroid hormone excess. Overall, both *Mct8KO* and *Mct8D1D2KO* mice have a mixed phenotype of age-dependent skeletal thyroid hormone deficiency and excess that is more severe in *Mct8D1D2KO* mutants.

### Role of circulating and local thyroid status in Mct8KO mice

Tissue thyroid hormone deficiency during skeletal development in *Mct8KO* mice occurs despite an elevated circulating T3 concentration and increased DIO1- and DIO2-mediated 5′-deiodination (19). Thus, tissue thyroid hormone deficiency during bone development in *Mct8KO* mice reflects impaired thyroid hormone entry into chondrocytes with an inadequate compensatory increase in local generation of T3 in the growth plate by DIO2. This problem appears critical at the time of maximal postnatal linear growth, which correlates with the normal peak in circulating thyroid hormone levels and represents a period of exquisite T3 sensitivity in growth plate chondrocytes (1, 25).

By contrast, tissue thyroid hormone excess in the adult skeleton in *Mct8KO* mice likely results from the moderate but persistently increased concentration of circulating T3. Bone turnover and remodeling are regulated by osteocytes, which orchestrate the balanced activities of bone-resorbing osteoclasts and bone-forming osteoblasts (33, 34), both of which express *Mct8* (4). Furthermore, *Dio2* has an essential homeostatic role in osteoblasts to optimize bone formation, mineralization, and strength (8). Thus, the phenotype of skeletal thyroid hormone excess in adult *Mct8KO* mice indicates an additional thyroid hormone transporter is expressed in osteocytes and/or osteoclasts and/or osteoblasts.

Taken together, these data demonstrate that MCT8 is an essential thyroid hormone transporter in growth plate chondrocytes, but its role in other skeletal cells is less critical.

### Role of circulating and local thyroid status in Mct8D1D2KO mice

Delayed skeletal development in *Mct8D1D2KO* mice occurs despite elevated circulating T3 levels throughout growth. Tissue thyroid hormone deficiency during endochondral ossification, and an absence of local T4 to T3 conversion in the skeleton of *Mct8D1D2KO* mice, indicate that *Mct8* is a major thyroid hormone transporter during linear growth. The similar defects in endochondral ossification observed in *Mct8KO* and *Mct8D1D2KO* mice (Figs. 2 and 3) further indicate that *Mct8* is functionally indispensable in growth plate chondrocytes. By contrast, the more severe osteoporotic phenotype in adult *Mct8D1D2KO* mice (Figs. 4–8) results at least in part from their prolonged prior exposure to elevated T3 (Fig. 1) (19), confirming that an additional transporter can compensate for *Mct8* deficiency in the regulation of bone mass and strength. Nevertheless, *Mct8D1D2KO* mice also have higher systemic T4 concentrations at all ages compared with *Mct8KO* mice, suggesting that the increased T4 levels may contribute to the more severe skeletal phenotype possibly via nongenomic actions (1).

### Increased TSH concentrations in Mct8KO and Mct8D1D2KO mice

A further consideration is that circulating TSH concentrations were increased by twofold to threefold in *Mct8KO* mice and by 100-fold in *Mct8D1D2KO* mice. Studies in TSH receptor (*Tshr*) KO mice led to the proposal that TSH acts as a negative regulator of bone remodeling (35). Although *Tshr* expression is well documented in osteoblasts, data from several laboratories are contradictory, demonstrating that TSH inhibits (35), has no effect on (36–38), or stimulates (39–41) osteoblast differentiation and activity. *Tshr* is also expressed in osteoclasts, and most studies indicate TSH inhibits osteoclastogenesis and function (35, 37, 41, 42). Overall, the proposal that TSH inhibits bone turnover and remodeling (35) has been supported by intervention studies in which intermittent treatment of ovariectomized rodents with TSH increased bone mass and strength (43).

In the current studies, however, both *Mct8KO* and *Mct8D1D2KO* mice have elevated circulating TSH levels but display an osteoporotic phenotype that is more severe in *Mct8D1D2KO* mice, in which TSH levels are much higher. These findings are not consistent with a major inhibitory role for TSH on bone turnover (35, 42, 43) in *Mct8* mutant mice. Rather, the findings demonstrate the fundamental importance of local temporal control of tissue T3 availability and action by thyroid hormone transporters and *Dio2* in bone.

### A physiological requirement for thyroid hormone transport in the skeleton

In conclusion, the current studies demonstrate a key role for MCT8 in growth plate chondrocytes during endochondral ossification and postnatal growth. The more severe bone loss in adult *Mct8D1D2KO* mice indicates an additional thyroid hormone transporter may act with MCT8 to regulate bone turnover. Overall, the studies demonstrate an essential physiological role for MCT8 in bone. MCT8 is a physiologically important thyroid hormone transporter in chondrocytes, but it is likely to act with an additional transporter in other skeletal cells to regulate the effects of T3 on adult bone mineralization, mass, and strength.
